# Presence of *B*. *thailandensis* and *B*. *thailandensis* expressing *B*. *pseudomallei*-like capsular polysaccharide in Thailand, and their associations with serological response to *B*. *pseudomallei*

**DOI:** 10.1371/journal.pntd.0006193

**Published:** 2018-01-24

**Authors:** Viriya Hantrakun, Janjira Thaipadungpanit, Patpong Rongkard, Prapaporn Srilohasin, Premjit Amornchai, Sayan Langla, Mavuto Mukaka, Narisara Chantratita, Vanaporn Wuthiekanun, David A. B. Dance, Nicholas P. J. Day, Sharon J. Peacock, Direk Limmathurotsakul

**Affiliations:** 1 Mahidol-Oxford Tropical Medicine Research Unit, Faculty of Tropical Medicine, Mahidol University, Bangkok, Thailand; 2 Centre for Tropical Medicine and Global Health, Nuffield Department of Clinical Medicine, Old Road Campus, University of Oxford, Oxford, United Kingdom; 3 Department of Microbiology and Immunology, Faculty of Tropical Medicine, Mahidol University, Bangkok, Thailand; 4 London School of Hygiene and Tropical Medicine, London, United Kingdom; 5 Lao-Oxford-Mahosot Hospital-Wellcome Trust Research Unit, Microbiology Laboratory, Mahosot Hospital, Vientiane, Lao People's Democratic Republic; 6 Department of Medicine, University of Cambridge, Cambridge, United Kingdom; 7 Department of Tropical Hygiene, Faculty of Tropical Medicine, Mahidol University, Bangkok, Thailand; Colorado State University, UNITED STATES

## Abstract

**Background:**

*Burkholderia pseudomallei* is an environmental Gram-negative bacillus and the cause of melioidosis. *B*. *thailandensis*, some strains of which express a *B*. *pseudomallei*-like capsular polysaccharide (BTCV), is also commonly found in the environment in Southeast Asia but is considered non-pathogenic. The aim of the study was to determine the distribution of *B*. *thailandensis* and its capsular variant in Thailand and investigate whether its presence is associated with a serological response to *B*. *pseudomallei*.

**Methodology/principal findings:**

We evaluated the presence of *B*. *pseudomallei* and *B*. *thailandensis* in 61 rice fields in Northeast (n = 21), East (n = 19) and Central (n = 21) Thailand. We found BTCV in rice fields in East and Central but not Northeast Thailand. Fourteen fields were culture positive for *B*. *pseudomallei* alone, 8 for *B*. *thailandensis* alone, 11 for both *B*. *pseudomallei* and *B*. *thailandensis*, 6 for both *B*. *thailandensis* and BTCV, and 5 for *B*. *pseudomallei*, *B*. *thailandensis* and BTCV. Serological testing using the indirect hemagglutination assay (IHA) of 96 farmers who worked in the study fields demonstrated that farmers who worked in *B*. *pseudomallei*-positive fields had higher IHA titers than those who worked in *B*. *pseudomallei*-negative fields (median 1:40 [range: <1:10–1:640] vs. <1:10 [range: <1:10–1:320], p = 0.002). In a multivariable ordered logistic regression model, IHA titers were significantly associated with the presence of *B*. *pseudomallei* (aOR = 3.7; 95% CI 1.8–7.8, p = 0.001) but were not associated with presence of *B*. *thailandensis* (p = 0.32) or BTCV (p = 0.32). One sequence type (696) was identified for the 27 BTCV isolates tested.

**Conclusions/significance:**

This is the first report of BTCV in Thailand. The presence of *B*. *pseudomallei* and *B*. *thailandensis* in the same field was not uncommon. Our findings suggest that IHA positivity of healthy rice farmers in Thailand is associated with the presence of *B*. *pseudomallei* in rice fields rather than *B*. *thailandensis* or BTCV.

## Author summary

*Burkholderia thailandensis* is a non-pathogenic soil-dwelling bacterium and is genetically closely related to *Burkholderia pseudomallei*, the cause of melioidosis. In mouse models, inoculation of a variant of *B*. *thailandensis* which express a *B*. *pseudomallei*-like capsular polysaccharide (BTCV) induces antibodies and partial protection against melioidosis. Here, we evaluated the presence of *B*. *pseudomallei*, *B*. *thailandensis*, and BTCV in 61 rice fields in Northeast, East and Central Thailand, determined whether they co-existed, and if their presence was associated with a serological response in farmers. We report the presence of BTCV in Thailand for the first time and describe the distribution of the three organisms. Co-localization between these organisms in the same rice fields was not uncommon. Our findings suggested that serological positivity based on the indirect hemagglutination assay (a test commonly used to detect antibodies to *B*. *pseudomallei*) in healthy rice farmers in Thailand was associated with exposure to *B*. *pseudomallei*, but not exposure to *B*. *thailandensis* or BTCV.

## Introduction

*Burkholderia pseudomallei* is a soil-dwelling Gram-negative bacterium and the cause of melioidosis, a frequently fatal infectious disease of humans and animals. Humans acquire the disease following skin inoculation, inhalation or ingestion of the bacterium from the environment. The disease is highly endemic in Southeast Asia and Northern Australia [1], and is increasingly being reported in South Asia, Africa and Central and South America [2, 3]. A recent modeling study estimated that there are about 165,000 human melioidosis cases per year worldwide, of whom 89,000 (54%) die [4]. The current diagnostic standard for melioidosis is microbiological culture [5]. However, melioidosis is difficult to diagnose due to its diverse clinical manifestations, the inadequacy of conventional bacterial identification methods, and a lack of microbiology laboratories in tropical developing countries [5]. An indirect haemagglutination assay (IHA) is the most frequently used serological test for melioidosis, but may be misleading when used for the diagnosis of melioidosis in disease-endemic regions [5]. This is because the background seropositivity (IHA titers ≥1:160) ranges from 4% to 32% in healthy individuals living in areas where melioidosis is endemic [6–8]. Therefore, IHA is recommended as a serological standard to assess exposure to *B*. *pseudomallei* [5].

*Burkholderia thailandensis* was first recognized by Wuthiekanun et al. in 1996 [9]. The organism is genetically closely related to *B*. *pseudomallei*, can be isolated from environmental soil and water, and is non-pathogenic [9–11]. The colony morphology of *B*. *thailandensis* and *B*. *pseudomallei* are very similar, but *B*. *thailandensis* can assimilate L-arabinose [12, 13]. In addition, *B*. *thailandensis* has polysaccharide-related genes that are distinct from *B*. *pseudomallei* (74.8% and 72.8% nucleotide and protein similarity, respectively) and usually lacks the virulence-associated capsular polysaccharide (also referred to as CPS or CPS-I) of *B*. *pseudomallei* [14–17]. The geographical distribution of *B*. *thailandensis* is uncertain but the organism has rarely been isolated from fields that are culture positive for *B*. *pseudomallei* [18, 19]. It was recently shown that *B*. *pseudomallei* can inhibit the growth and motility of *B*. *thailandensis* in the laboratory [20]. However, previous environmental studies did not systematically evaluate the presence of both organisms, so the presence of *B*. *thailandensis* and co-localization of both organisms may have been underestimated [18, 19]. In an experimental mouse model, lipopolysaccharide extracted from *B*. *thailandensis* induced measurable IgG and IgM, and provided partial protection against melioidosis [21]. The association between exposure to environmental *B*. *thailandensis* and IHA seropositivity in humans is still largely unknown.

A variant of *B*. *thailandensis* originally isolated from soil in Cambodia (E555) that contained genes encoding a *B*. *pseudomallei*-like capsular polysaccharide cluster (BTCV) was described in 2010 [17]. This organism exhibited several *B*. *pseudomallei*-like phenotypes including colony wrinkling, resistance to human complement binding, and intracellular macrophage survival. However, in mice E555 was avirulent [17], induced higher levels of IgG and gave better protection against melioidosis than non-capsulated *B*. *thailandensis* [22]. The capsular polysaccharide (CPS) biosynthesis gene cluster of E555 and that of *B*. *pseudomallei* are highly similar (94.4% and 96% nucleotide and protein similarity, respectively) [17], and nuclear magnetic resonance spectroscopy has shown that the structures of CPS produced by E555 and that of *B*. *pseudomallei* are identical [23]. Previously, BTCV has been isolated from human blood in the USA in 2003 (strain CDC3015869; ST101, USA [24]) and from environmental samples in Cambodia in 2010 (strain E555; ST696), Gabon in 2013 (strain D50; ST1126 [25]) and Laos in 2015 (strain ST_10; ST696 [26]). BTCV has not been reported in Thailand and its distribution is unknown.

We recently reported the presence of *B*. *pseudomallei* in 61 rice fields in the Northeast, East and Central Thailand, and its association with soil physicochemical properties [27]. Here, we report the presence of *B*. *thailandensis* and co-localization between *B*. *pseudomallei* and *B*. *thailandensis* in the same rice fields, and provide the first report of the BTCV in Thailand. In addition, we explored whether exposure to *B*. *thailandensis* and BTCV is associated with background seropositivity to *B*. *pseudomallei* by evaluating IHA levels in healthy adults who worked in the sampled rice fields.

## Materials and methods

### Study area

East, Central and Northeast Thailand consist of 7, 21 and 20 provinces, cover 34,381, 93,005 and 168,854 km^2^, and had estimated populations in 2013 of 3.9, 18.7 and 23.3 million, respectively [28]. Northeast Thailand is a plateau surrounded by mountain ranges, and most of the arable land consists of tropical sandy soil. East Thailand is characterized by short mountain ranges alternating with alluvial plains. Central Thailand is a large plain consisting of clay soil. Rice farming is the predominant form of agriculture in all three regions. In Thailand, for administrative purposes each province is sub-divided into districts, sub-districts, communes and villages. The majority of the population in all three regions live in rural settings and most adults are engaged in agriculture, particularly rice farming. In 2013, areas used for agriculture were 57%, 48% and 60% in East, Central and Northeast Thailand, respectively [29].

### Study design

We conducted a cross-sectional environmental survey as described previously [27]. All *B*. *pseudomallei*, *B*. *thailandensis* and BTCV reported in this work were from the same environmental survey [27]. In brief, we collected soil from randomly-selected rice fields in the East, Central and Northeast regions during the dry season (from April–June) in 2013, 2014 and 2015, respectively. We sampled rice fields that had been used for rice farming in the 12 months prior to the sampling date. We collected the blood from farmers who were exposed to the sampled rice fields in the 12 months prior to the blood collection date.

### Ethics statement

Written, informed consent was obtained from land owners and farmers prior to soil sampling and blood collection, respectively. The study protocol was approved by the Ethics Committee of the Faculty of Tropical Medicine, Mahidol University (MUTM 2013-021-01) and the Oxford Tropical Research Ethics Committee, University of Oxford (OXTREC 1013–13).

### Soil sampling and soil properties

The method used for soil sampling was described previously [27, 30]. In brief, each rice field was divided into a 10x10 grid system to generate 100 sampling points per field. At each sampling point, around 30 grams of soil was removed from the base of a 30-cm hole. All soil samples were processed within 48 hours of collection for the identification of *B*. *pseudomallei* and *B*. *thailandensis*, and for soil physicochemical properties. One kilogram of soil from each sampling field was made by aggregating 100 soil samples (10 g per each sampling point) and evaluated for soil physicochemical properties as described previously [27].

### Identification of *B*. *pseudomallei* and *B*. *thailandensis*

Ten grams of soil from each sampling point was mixed with 10 ml of enrichment broth consisting of threonine-basal salt solution plus colistin 50mg/L (TBSS-C50 broth) and incubated at 40°C in air for 48 hours. Ten microliters of surface liquid was then streaked onto Ashdown agar containing gentamicin 8mg/L and crystal violet 5mg/L using a calibrated loop, incubated at 40°C in air, and examined every day for 4 days for bacterial colonies suggestive of *B*. *pseudomallei* or *B*. *thailandensis* [31, 32]. *B*. *pseudomallei* can have seven colony morphotypes on Ashdown agar [32], and cannot readily be distinguished from the colonies of *B*. *thailandensis* [9, 12, 13]. For each soil specimen, a total of up to five presumptive colonies of *B*. *pseudomallei* or *B*. *thailandensis* were picked and evaluated by a latex agglutination test that is highly specific for *B*. *pseudomallei* CPS [16, 33, 34], and the L-arabinose assimilation test. *B*. *pseudomallei* was defined based on a positive latex agglutination test and negative L-arabinose assimilation. *B*. *thailandensis* was defined based on a negative latex agglutination test and positive L-arabinose assimilation [9, 16, 17]. Colonies which had positive results for both the latex agglutination and L-arabinose assimilation tests were defined as BTCV, as previously described [17]. The monoclonal antibody used in the latex agglutination test has been shown to be positive for *B*. *pseudomallei* and BTCV (strains E555 and CDC3015869), and negative for *B*. *thailandensis* [35, 36].

### Genotyping of BTCV

Because BTCV was found in rice fields in East and Central Thailand, we randomly selected three isolates of BTCV from each culture positive field and genotyped these using multilocus sequence typing (MLST) [37]. The alleles at each of the seven loci were assigned by comparing the sequences to those on the *B*. *pseudomallei* MLST website (https://pubmlst.org/bpseudomallei/).

Information and the sequence type of BTCV reported in this work has been deposited in the global MLST database (https://pubmlst.org/bpseudomallei/).

### Serological response

Blood samples were collected from rice farmers who had worked in the sampled rice fields in the 12 months prior to the sampling date. The detection of antibodies against *B*. *pseudomallei* was performed using the IHA, as described previously [38, 39]. The antigen used in the IHA was derived from a pool of two clinical *B*. *pseudomallei* isolates, 199a and 207a, obtained from melioidosis patients in Ubon Ratchathani, Thailand. The negative control was pooled sera from three patients with no detectable IHA titers. The positive control was pooled sera from three patients with known positive IHA. In this study, for binary comparisons, an IHA titer of <1:80 was defined as negative based on the previous report that healthy US donor could have IHA titers up to 1:40 [40].

### Sample size calculation

To determine the optimal sample size, we performed a pilot study of soil sampling in four rice fields in Chachoengsao province, East Thailand. Three and four rice fields were culture positive for *B*. *pseudomallei* (75%; 3 of 4) and *B*. *thailandensis* (100%; 4 of 4), respectively. We calculated that 60 rice fields (3 rice fields per province) and 60 rice farmers were needed to determine environmental factors associated with presence of *B*. *pseudomallei* and *B*. *thailandensis*, and evaluate the association between IHA titers and presence of both organisms, respectively, with a power of 80% at an alpha error of 5%.

### Statistical analysis

We evaluated (1) positivity of *B*. *thailandensis* and BTCV in the rice fields, (2) co-localization and correlation between *B*. *pseudomallei*, *B*. *thailandensis* and BTCV in the rice fields, (3) soil properties associated with presence of *B*. *thailandensis* and BTCV, and (4) association between IHA levels and presence of those organisms in the rice fields. Fisher’s exact test and Mann-Whitney test were performed to compare binary and ordinal variables, respectively. McNemar’s test was used to compare the presence of two organisms. We assessed co-localization between organisms in the rice fields by using Kappa value. The Kappa value was used to describe the agreement of presence and absence of the organism in rice fields, beyond that caused by chance, as follows: 0.00–0.20, slight; 0.21–0.40, fair; 0.41–0.60, moderate; 0.61–0.80, substantial; 0.81–1.00, high [41]. A Spearman rank correlation coefficient (Spearman’s rho) was used to assess correlations among the total number of sampling points culture-positive for *B*. *pseudomallei*, *B*. *thailandensis* and BTCV in the rice fields. Spearman’s rho close to 0 indicates no correlation, while Spearman’s rho close to 1 (or -1) indicates a strong positive (or negative) correlation between the organisms [42]. We used ordered logistic regression to determine the associations between IHA level and presence of the three organisms. As we sampled more than one rice farmer per field, the analysis was stratified by rice field. Multivariable ordered logistic regression models were developed using a purposeful selection method [43]. In brief, a univariable ordered logistic regression model was used to preliminarily evaluate the crude association between presence of *B*. *pseudomallei*, *B*. *thailandensis* and BTCV and IHA titers. We decided *a priori* to evaluate their independent associations in a multivariable ordered logistic regression model, and presence of *B*. *pseudomallei*, *B*. *thailandensis* and BTCV were all included in the final multivariable model. Sensitivity analysis was conducted by grouping *B*. *thailandensis* and BTCV as *B*. *thailandensis*. All statistical tests were performed using STATA version 14.0 (StataCorp LP, College Station, Texas). The final database with the data dictionary are publicly available online (https://doi.org/10.6084/m9.figshare.4928993).

## Results

### Distribution of *B*. *thailandensis* and BTCV

**Fig 1:**
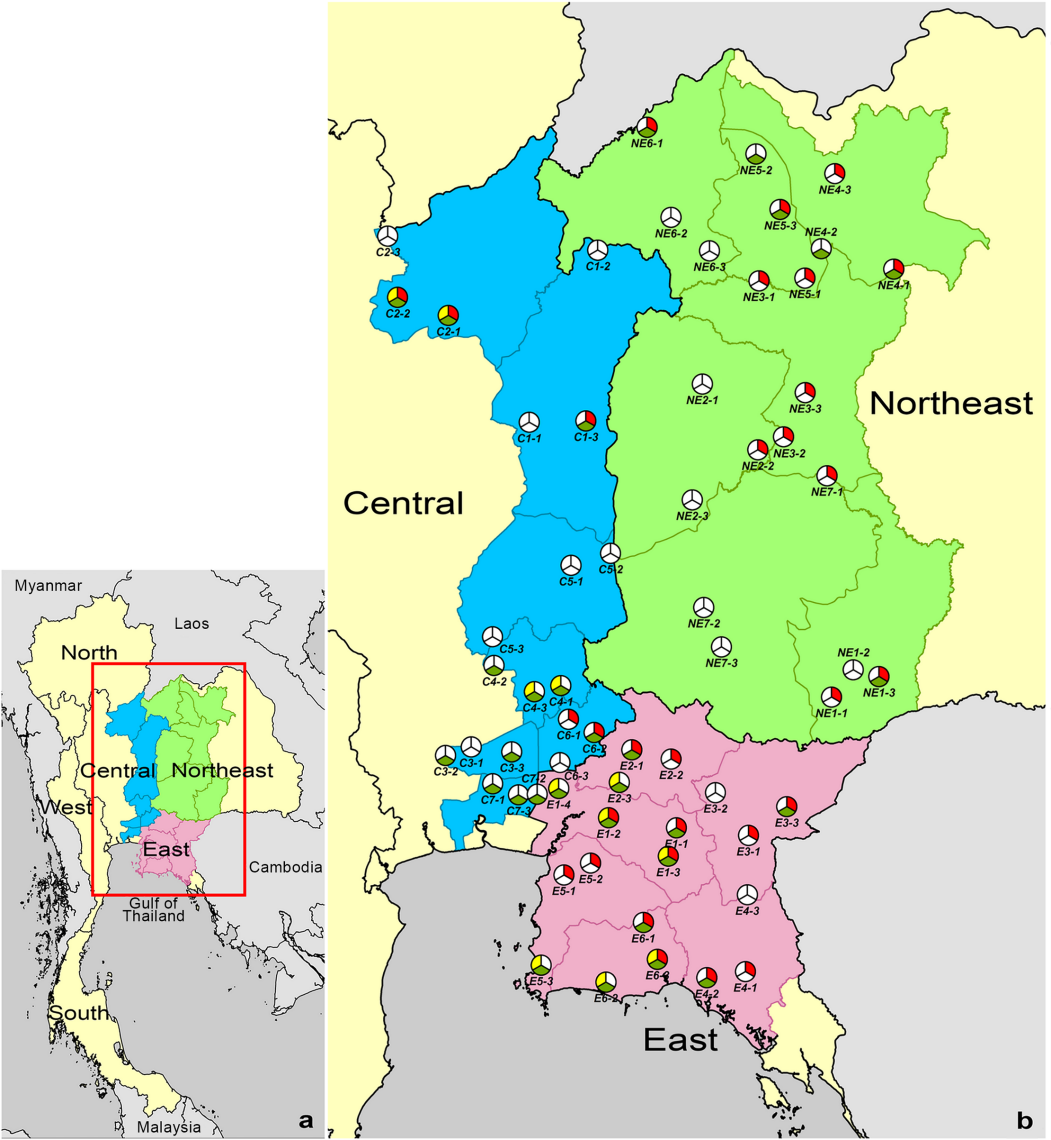
Map of the presence of *B*. *pseudomallei*, *B*. *thailandensis* and *B*. *thailandensis* expressing *B*. *pseudomallei*-like capsular polysaccharide (BTCV) in 61 rice fields in Northeast (n = 21), East (n = 19) and Central (n = 21) Thailand. (a) Map of Thailand. (b) Location of the 61 rice fields evaluated. Red, green and yellow pies represent rice fields that were culture positive and negative for *B*. *pseudomallei*, *B*. *thailandensis*, and BTCV, respectively. Province codes represent Burirum (NE1), Chaiyaphum (NE2), Khon Kaen (NE3), Udon Thani (NE4), Nong Bua Lam Phu (NE5), Loei (NE6) and Nakhon Ratchasima (NE7) in the Northeast, Chachoengsao (E1), Prachinburi (E2), Sa Kaeo (E3), Chanthaburi (E4), Chonburi (E5) and Rayong (E6) in the East, Phetchabun (C1), Phitsanulok (C2), Pathum Thani (C3), Saraburi (C4), Lopburi (C5), Nakhon Nayok (C6) and Bangkok (C7) in Central Thailand. ArcGis Version 10.2 (ESRI, Redlands, CA, USA) was used to map the sampled rice fields. The location of sampled rice fields was recorded by using the EpiCollect application (www.epicollect.net, Imperial College, London).

Of 6,100 soil samples collected from 61 rice fields, 826 (14%) were culture positive for *B*. *thailandensis*. The percentages of rice fields that were culture-positive for *B*. *thailandensis* were 29% (6 of 21 rice fields), 63% (12 of 19 rice fields) and 57% (12 of 21 rice fields) in Northeast, East and Central Thailand, respectively (Fig 1). There was borderline evidence that culture-positivity for *B*. *thailandensis* was higher in the East than the Northeast (63% vs. 29%, p = 0.055), while there was no significant difference between East and Central (63% vs. 57%, p = 0.76) or Northeast and Central regions (29% vs. 57%, p = 0.12).

For the rice fields that were culture-positive for *B*. *thailandensis*, the median numbers of positive sampling points were 6.5 (range 1 to 27), 26.5 (range 1 to 100) and 10.5 (range 1 to 85) in Northeast, East and Central Thailand, respectively (S1 Table). There was a trend towards the median number of positive sampling points for *B*. *thailandensis* being lower in the Northeast than the East (p = 0.08), while there was no significant difference between Central versus Northeast or East Thailand (p = 0.40 and 0.52, respectively).

BTCV was isolated from 11 of 61 (18%) rice fields in the East (7 fields) and Central (4 fields) regions but it was not isolated from rice fields in Northeast Thailand (Fig 1). Overall, the proportion of fields positive for the BTCV was lower than that for *B*. *thailandensis* (18% vs. 49%, p<0.001). The percentage of rice fields that were culture positive for BTCV in the East and Central regions was not significantly different (37% [7/19] vs. 19% [4/21], p = 0.29). The median numbers of positive sampling points in the East and Central regions were also not significantly different (3 [range 1 to 24] vs. 4 [range 1 to 8], p = 0.45).

### Co-localization and correlation between presence of *B*. *pseudomallei*, *B*. *thailandensis* and BTCV in the same rice fields

**Fig 2:**
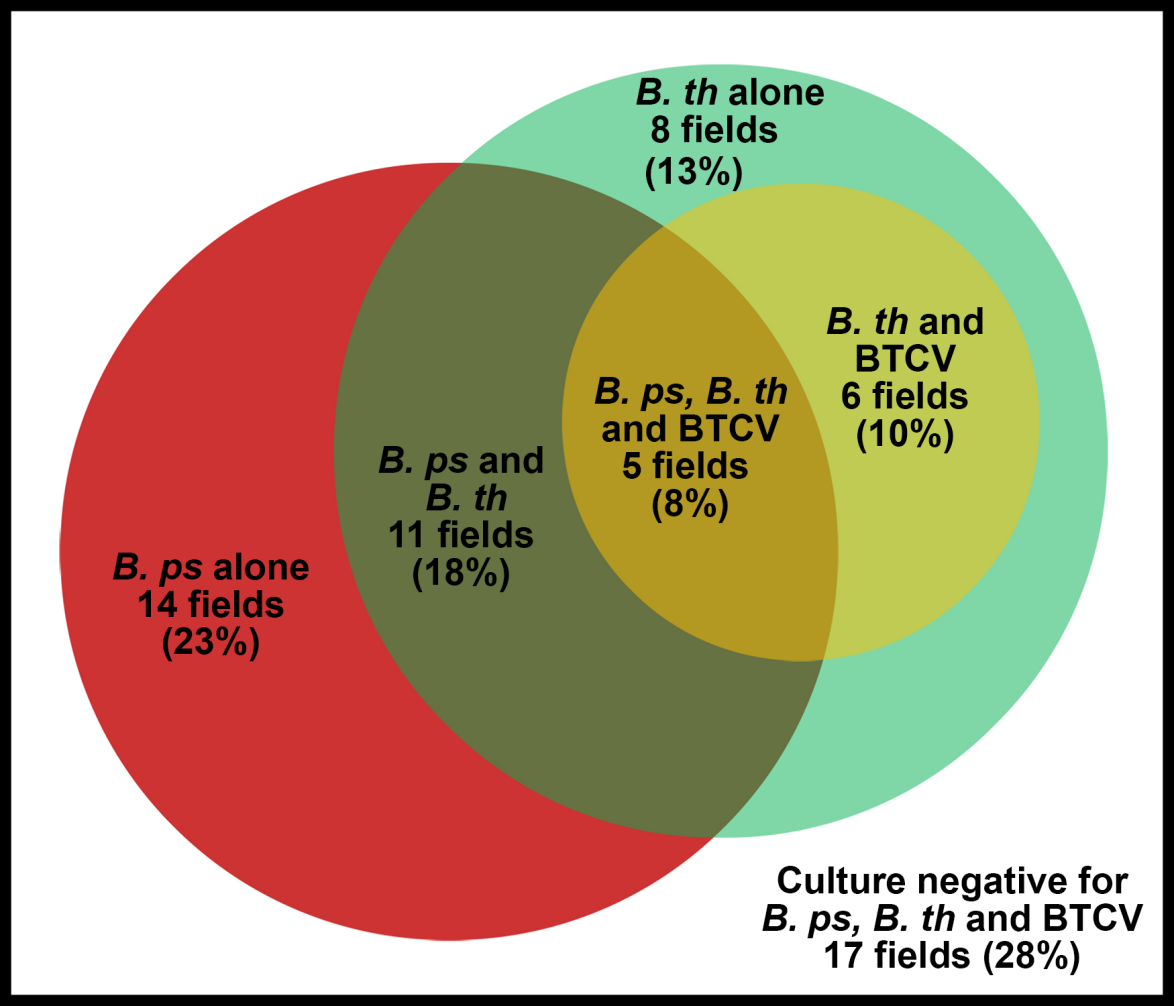
Overlap between presence of *B*. *pseudomallei* (*B*. *ps*; red), *B*. *thailandensis* (*B*. *th*; green) and *B*. *thailandensis* expressing *B*. *pseudomallei*-like capsular polysaccharide (BTCV; yellow) in 61 sampled rice fields.

We previously reported the isolation of *B*. *pseudomallei* from 30 of 61 rice fields included in this study [27]. Fig 2 shows the number of rice fields from which *B*. *pseudomallei*, *B*. *thailandensis* and BTCV were isolated. Of 61 rice fields, 14 (23%) were positive for *B*. *pseudomallei* alone, 8 (13%) positive for *B*. *thailandensis* alone, 11 (18%) positive for both *B*. *pseudomallei* and *B*. *thailandensis*, 6 (10%) positive for *B*. *thailandensis* and BTCV, and 5 (8%) positive for *B*. *pseudomallei*, *B*. *thailandensis* and BTCV. Co-localization of *B*. *pseudomallei* and *B*. *thailandensis* in the same rice field was not more frequent than expected by chance (Kappa value 0.08, p = 0.26). The numbers of sampling points per rice field that were culture positive for *B*. *pseudomallei* and for *B*. *thailandensis* were not correlated (Spearman’s rho -0.02, 95%CI -0.27 to 0.23, p = 0.89). A sensitivity analysis was conducted by considering BTCV as *B*. *thailandensis*, which gave a comparable result (Spearman’s rho -0.02, 95%CI -0.27 to 0.23, p = 0.87). All eleven fields culture positive for BTCV were also culture positive for *B*. *thailandensis* (Fig 2). There was a fair agreement between presence of *B*. *thailandensis* and BTCV (Kappa value 0.37, p<0.001), and a strong correlation between the total number of sampling points culture positive for the two organisms (Spearman’s rho 0.68, 95% CI 0.51 to 0.79, p<0.001).

### Co-localization and correlation between presence of *B*. *pseudomallei*, *B*. *thailandensis* and BTCV in the same sampling points

Of 6,100 soil samples collected, 975 (16%) were positive for *B*. *pseudomallei* alone, 706 (12%) positive for *B*. *thailandensis* alone, 24 (0.4%) positive for BTCV alone, 69 (1%) positive for both *B*. *pseudomallei* and *B*. *thailandensis*, 1 (0.02%) positive for *B*. *pseudomallei* and BTCV, 50 (0.8%) positive for *B*. *thailandensis* and BTCV, and 1 (0.02%) positive for *B*. *pseudomallei*, *B*. *thailandensis* and BTCV. There was a slight agreement between presence of *B*. *thailandensis* and BTCV in the same soil sample (Kappa value 0.09, p<0.001). Co-localization of *B*. *pseudomallei* and *B*. *thailandensis* in the same soil sample was also not greater than that expected by chance (p>0.99).

### Soil physicochemical properties associated with presence of *B*. *thailandensis* and BTCV

Associations between soil physicochemical properties and the presence of *B*. *thailandensis* were not observed (S2 Table). Presence of BTCV was negatively associated with cation exchange capacity, which represents the total nutrient fixing capacity of soil (p = 0.05), and associated with the level of total nitrogen (p = 0.04; S3 Table). The associations were also observed in the multivariable model (S4 Table).

### Genetic diversity of BTCV

A total of 27 isolates of BTCV from 76 culture positive sampling points for BTCV in 11 rice fields in East and Central Thailand were randomly selected for MLST (S5 Table). All 27 isolates belonged to sequence type (ST) 696, which was identical to the ST of BTCV strain E555 reported from soil in Cambodia [17]. We had previously reported a single *B*. *pseudomallei* isolate (strain A-330-05-1-04) from drinking water in Ubon Ratchathani, northeast Thailand, as ST696 [44]. The isolate was re-evaluated. The isolate was found to be positive for both latex agglutination and L-arabinose assimilation and was thus re-classified as BTCV.

### IHA titers and their association with presence of *B*. *pseudomallei*, *B*. *thailandensis* and BTCV

**Fig 3:**
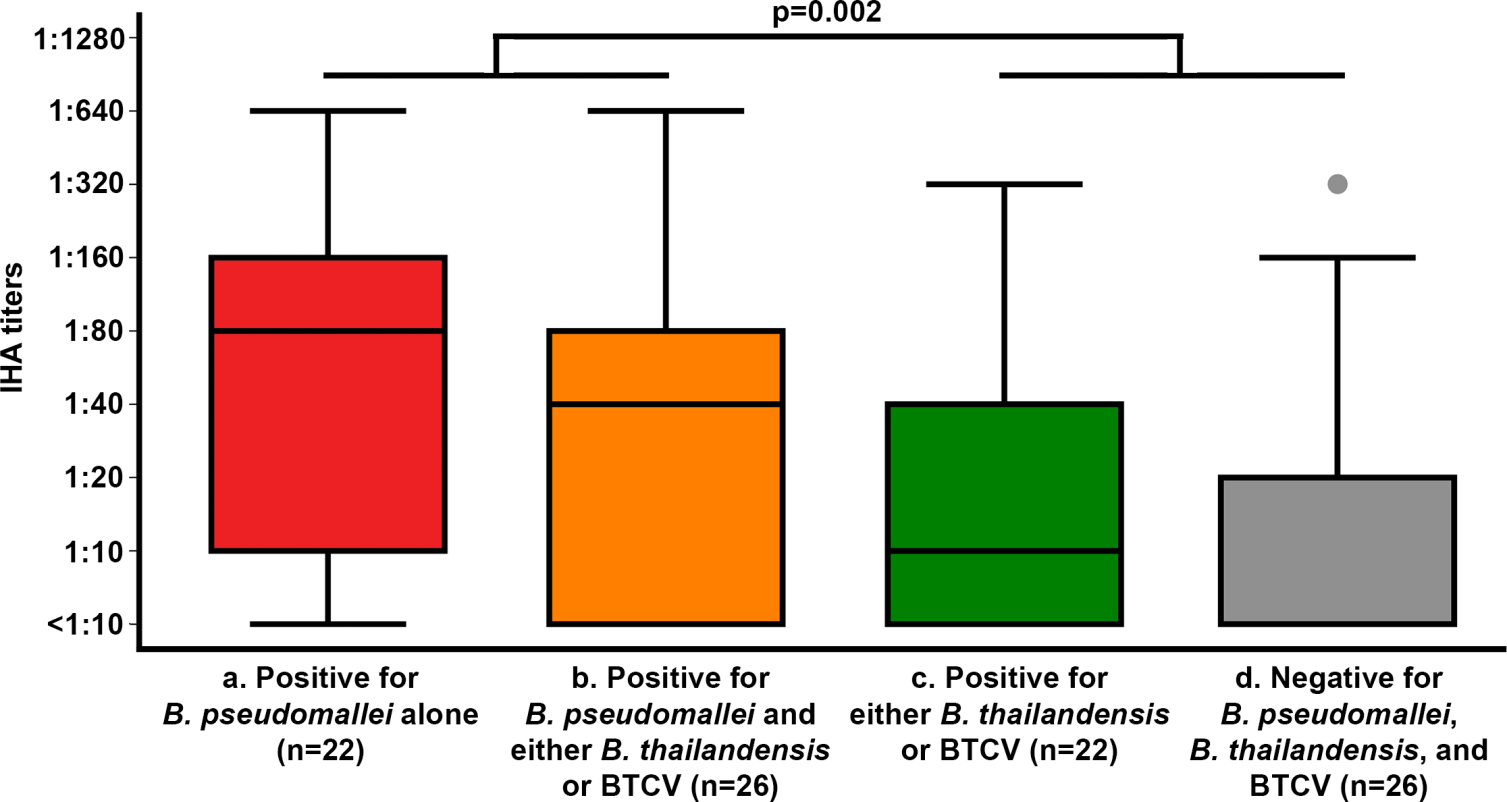
IHA titers associated with the presence of *B*. *pseudomallei*, *B*. *thailandensis* and *B*. *thailandensis* expressing *B*. *pseudomallei*-like capsular polysaccharide (BTCV) in the rice fields, respectively. Box-and-whisker plots indicate median, interquartile range and distribution of IHA titers. Dots indicate outliers (data located outside 1.5 times of interquartile range). (a) IHA titers of farmers whose rice fields were culture positive for *B*. *pseudomallei* alone (22 farmers). (b) IHA titers of farmers whose rice fields were culture positive for *B*. *pseudomallei* and either *B*. *thailandensis* or BTCV (26 farmers), (c) IHA titers of farmers whose rice fields culture positive for either *B*. *thailandensis* or BTCV (22 farmers), and (d) IHA titers of farmers whose rice fields culture negative for *B*. *pseudomallei*, *B*. *thailandensis* and BTCV (26 farmers).

Of 96 rice farmers included in the analysis, 29, 35 and 32 were from Northeast, East and Central Thailand, respectively. The median number of farmers per rice field was 1 (range 1 to 5). Sixty-two farmers (65%) were male and median age was 51 years (range 23–75 years). Six farmers (6%) had a known diagnosis of diabetes. Overall, 27 (28%) farmers had a positive IHA (IHA titers ≥1:80). Forty eight farmers who worked in rice fields culture-positive for *B*. *pseudomallei* had higher IHA titers than the 48 farmers who worked in rice fields culture-negative for the organism (median 1:40 [range: <1:10–1:640] vs. <1:10 [range: <1:10–1:320], p = 0.002) (Fig 3). Proportion of farmers who had positive IHA (IHA ≥1:80) was also significantly higher in rice field culture-positive for *B*. *pseudomallei* than the farmers who work in rice field culture-negative for *B*. *pseudomallei* (44% vs. 13%, p = 0.001).

In the univariable ordered logistic regression model, IHA titers were associated with the presence of *B*. *pseudomallei* (OR = 3.39; 95% CI 1.66–6.90, p = 0.001) but not with presence of *B*. *thailandensis* (OR = 0.92; 95% CI 0.44–1.91, p = 0.82) or BTCV (OR = 1.04; 95% CI 0.60–1.80, p = 0.89). A multivariable ordered logistic regression model was used to evaluate independent association between IHA titers of rice farmers and presence of each organism. IHA titers were independently associated with the presence of *B*. *pseudomallei* (aOR = 3.72; 95% CI 1.76–7.84, p = 0.001) but not associated with the presence of *B*. *thailandensis* (p = 0.32) or BTCV (p = 0.32, Table 1).

**Table 1:** Factors associated with indirect hemagglutination assay (IHA) results in 96 healthy rice farmers.

Organisms cultured from rice fields	IHA results		Odds Ratios (95% confidence interval)*	
	IHA positive ** (n = 27 famers)	IHA negative ** (n = 69 farmers)	Univariable analysis	Multivariable analysis
***B*. *pseudomallei***	21/27 (78%)	27/69 (39%)	3.39 (1.66–6.90), p = 0.001	3.72 (1.76–7.84), p = 0.001
***B*. *thailandensis***	12/27 (44%)	36/69 (52%)	0.92 (0.44–1.91), p = 0.82	0.63 (0.25–1.57), p = 0.32
**BTCV**	4/27 (15%)	17/69 (25%)	1.04 (0.60–1.80), p = 0.89	1.62 (0.63–4.17), p = 0.32

* Estimated by ordered logistic regression models stratified by sampled rice field.

** IHA titers ≥ 1:80 is defined as positive; IHA titers <1:80 is defined as negative.

## Discussion

Here, we present data on the spatial distribution of *B*. *pseudomallei*, *B*. *thailandensis* and BTCV in Northeast, East and Central Thailand. This is the first study to report the isolation of BTCV from soil in Thailand, although we did find that a Thai water isolate previously identified as *B*. *pseudomallei* was actually an example of BTCV [44]. *B*. *thailandensis* was commonly isolated in all three regions, while BTCV was less common but associated with *B*. *thailandensis*. Co-localization of *B*. *thailandensis* and *B*. *pseudomallei* was not uncommon. Our findings also suggest that IHA positivity of healthy rice farmers was associated with exposure to *B*. *pseudomallei* rather than to *B*. *thailandensis* or BTCV. This supports the recommendation that IHA could be used to measure exposure to environmental *B*. *pseudomallei* [5], even in areas containing other closely related *Burkholderia* species.

Our finding of co-localization of *B*. *pseudomallei* and *B*. *thailandensis* is consistent with a previous environmental study in Khon Kaen, northeast Thailand [11]. *B*. *thailandensis* has been reported from many melioidosis-endemic countries; including Thailand, Laos, Vietnam [37], Cambodia (https://pubmlst.org/bpseudomallei/), Australia [45], Papua New Guinea [46], Kenya [37] and Gabon [25]. This is probably highly influenced by the locations of melioidosis research groups. *B*. *thailandensis* has also been reported in two non melioidosis-endemic countries; France and the United States [24, 37]. *B*. *thailandensis* strain 82172 (ST73) was isolated from the intestine of a foal in France in 1982, while *B*. *thailandensis* strain CDC2721121 (ST73) was isolated from the pleural wound of a 76 year old male from Louisiana, USA, in 1997 [24, 37]. As strain CDC2721121 was isolated from a wound sample its pathogenicity in humans cannot be assumed and this strain was avirulent in a mouse model [22].

Although BTCV was common in both Central and East Thailand, its prevalence is lower than that of wild-type *B*. *thailandensis*. Our analysis suggested that BTCV was associated with soil with low cation exchange capacity and high levels of total nitrogen. Although Sim *et al*. raised the possibility that acquisition of the *B*. *pseudomallei*-like CPS in E555 might improve its environmental fitness [17], this is not supported by our findings. It is also possible that the environment we studied is not representative of the environmental niche which induced *B*. *thailandensis* to acquire the *B*. *pseudomallei*-like gene cluster.

The finding that all BTCV isolates obtained from different geographical areas in Thailand, Laos and Cambodia were ST696 suggests that these may have arisen from a single ancestor. BTCV isolates in USA (ST101) [24] and Gabon (ST1126) [25] are single- and triple-locus variants of ST696, respectively (S5 Table). Previous phylogenetic analysis suggested that ST101 and ST696 are closely related and possibly share the same ancestor [17]. Studies using whole genome sequencing of BTCV and *B*. *thailandensis* from different regions are required to further understand the genetic diversity and evolution of this organism.

Our results suggest that exposure to environmental *B*. *thailandensis* and BTCV makes a limited contribution to IHA seropositivity in farmers. In animal models, antibodies can be detected after intraperitoneal inoculation of *B*. *thailandensis* and BTCV [22]. Intraperitoneal inoculation can lead to rapid dissemination of *B*. *thailandensis* or BTCV by bypassing natural host defenses [22], and induces a serological response. Nonetheless, human exposure to *B*. *thailandensis* and BTCV in the natural environment rarely if ever leads to infection, unlike exposure to *B*. *pseudomallei*. This is also supported by the finding that intra-nasal inoculation of high dose *B*. *thailandensis* or BTCV (10^6^ colony forming unit [CFU]) did not cause death in BALB/c mice with rapid bacterial clearance and no visible abscess formation in sacrificed mice, whilst the LD50 of *B*. *pseudomallei* was less than 300 CFU in the same experiment [17].

Our study has several limitations. Soil sampling was performed during the dry season over a period of three years. We chose to sample during the dry season to control the variation in presence of the three organisms, human exposure and soil physicochemical properties associated with seasonal changes. It is possible that the presence of *B*. *thailandensis* and BTCV could vary according to the season. Farmers may work in multiple rice fields, and be exposed to *B*. *pseudomallei* in untested fields. Our study may have also detected more positive samples for *B*. *thailandensis* and BTCV if more than five colonies had been tested from each sampling point or using other identification methods such as PCR.

In summary, our large cross-sectional environmental survey has defined the distribution of *B*. *thailandensis* and BTCV in Thailand. This is the first report of BTCV in Thailand, which appears to be less common than wild-type *B*. *thailandensis*. Our findings also suggest that exposure to *B*. *thailandensis* or BTCV in the environment makes a limited contribution to IHA positivity amongst healthy farmers.

## Supporting information

S1 Table Number of culture-positive sampling points for *B*. *pseudomallei* (*B*. *ps*), *B*. *thailandensis* (*B*. *th*) and *B*. *thailandensis* expressing *B*. *pseudomallei*-like capsular polysaccharide variant (BTCV) in 61 rice fields in the Northeast (n = 21), East (n = 19) and Central (n = 21) Thailand. (PDF)

S2 Table Soil physicochemical properties associated with the presence of *B*. *thailandensis* in univariable logistic regression models. (PDF)

S3 Table Soil physicochemical properties associated with the presence of BTCV in univariable logistic regression models. (PDF)

S4 Table Soil physicochemical properties associated with the presence of BTCV in a multivariable logistic regression model. (PDF)

S5 Table Reports of *B*. *thailandensis* with *B*. *pseudomallei*-like capsular polysaccharide worldwide from 1921 to 2016. (PDF)

## References

[1] Wiersinga WJ, Currie BJ, Peacock SJ. Melioidosis. N Engl J Med. 2012;367(11):1035–44. doi: 10.1056/NEJMra1204699 . 22970946 10.1056/NEJMra1204699

[2] Redondo MC, Gomez M, Landaeta ME, Rios H, Khalil R, Guevara RN, et al Melioidosis presenting as sepsis syndrome: a case report. Int J Infect Dis. 2011;15(3):e217–8. doi: 10.1016/j.ijid.2010.11.009 . 21195650 10.1016/j.ijid.2010.11.009

[3] Currie BJ, Dance DA, Cheng AC. The global distribution of *Burkholderia pseudomallei* and melioidosis: an update. Trans R Soc Trop Med Hyg. 2008;102 Suppl 1:S1–4. doi: 10.1016/S0035-9203(08)70002-6 . 19121666 10.1016/S0035-9203(08)70002-6

[4] Limmathurotsakul D, Golding N, Dance DAB, Messina JP, Pigott DM, Moyes CL, et al Predicted global distribution of *Burkholderia pseudomallei* and burden of melioidosis. Nature Microbiol. 2016;1:15008 doi: 10.1038/nmicrobiol.2015.8 10.1038/nmicrobiol.2015.8 27571754

[5] Hoffmaster AR, AuCoin D, Baccam P, Baggett HC, Baird R, Bhengsri S, et al Melioidosis diagnostic workshop, 2013. Emerg Infect Dis. 2015;21(2). doi: 10.3201/eid2102.141045 25626057 10.3201/eid2102.141045 PMC4313648

[6] Maude RR, Maude RJ, Ghose A, Amin MR, Islam MB, Ali M, et al Seroepidemiological surveillance of *Burkholderia pseudomallei* in Bangladesh. Trans R Soc Trop Med Hyg. 2012;106(9):576–8. doi: 10.1016/j.trstmh.2012.06.003 22795754 10.1016/j.trstmh.2012.06.003 PMC3424416

[7] Hii SYF, Kee CC, Ahmad N. Melioidosis: Overview of seropositivity in Malaysia. Trop Biomed. 2016;33(4):4. 33579066

[8] Suttisunhakul V, Wuthiekanun V, Brett PJ, Khusmith S, Day NP, Burtnick MN, et al Development of Rapid Enzyme-Linked Immunosorbent Assays for Detection of Antibodies to *Burkholderia pseudomallei*. J Clin Microbiol. 2016;54(5):1259–68. doi: 10.1128/JCM.02856-15 . 26912754 10.1128/JCM.02856-15 PMC4844749

[9] Wuthiekanun V, Smith MD, Dance DA, Walsh AL, Pitt TL, White NJ. Biochemical characteristics of clinical and environmental isolates of *Burkholderia pseudomallei*. J Med Microbiol. 1996;45(6):408–12. doi: 10.1099/00222615-45-6-408 . 8958243 10.1099/00222615-45-6-408

[10] Trakulsomboon S, Dance DA, Smith MD, White NJ, Pitt TL. Ribotype differences between clinical and environmental isolates of *Burkholderia pseudomallei*. J Med Microbiol. 1997;46(7):565–70. doi: 10.1099/00222615-46-7-565 . 9236740 10.1099/00222615-46-7-565

[11] Sermswan RW, Royros P, Khakhum N, Wongratanacheewin S, Tuanyok A. Direct detection of *Burkholderia pseudomallei* and biological factors in soil. Trans R Soc Trop Med Hyg. 2015;109(7):462–8. doi: 10.1093/trstmh/trv040 . 26048871 10.1093/trstmh/trv040

[12] Smith MD, Angus BJ, Wuthiekanun V, White NJ. Arabinose assimilation defines a nonvirulent biotype of *Burkholderia pseudomallei*. Infect Immun. 1997;65(10):4319–21. 9317042 10.1128/iai.65.10.4319-4321.1997 PMC175618

[13] Brett PJ, DeShazer D, Woods DE. Burkholderia thailandensis sp. nov., a *Burkholderia pseudomallei*-like species. Int J Syst Bacteriol. 1998;48 Pt 1:317–20. doi: 10.1099/00207713-48-1-317 . 9542103 10.1099/00207713-48-1-317

[14] Reckseidler SL, DeShazer D, Sokol PA, Woods DE. Detection of bacterial virulence genes by subtractive hybridization: identification of capsular polysaccharide of *Burkholderia pseudomallei* as a major virulence determinant. Infect Immun. 2001;69(1):34–44. doi: 10.1128/IAI.69.1.34-44.2001 11119486 10.1128/IAI.69.1.34-44.2001 PMC97852

[15] Smith MD, Wuthiekanun V, Walsh AL, Pitt TL. Latex agglutination test for identification of *Pseudomonas pseudomallei*. J Clin Pathol. 1993;46(4):374–5. 7684405 10.1136/jcp.46.4.374 PMC501225

[16] Wuthiekanun V, Anuntagool N, White NJ, Sirisinha S. Short report: a rapid method for the differentiation of *Burkholderia pseudomallei* and *Burkholderia thailandensis*. Am J Trop Med Hyg. 2002;66(6):759–61. . 12224587 10.4269/ajtmh.2002.66.759

[17] Sim BM, Chantratita N, Ooi WF, Nandi T, Tewhey R, Wuthiekanun V, et al Genomic acquisition of a capsular polysaccharide virulence cluster by non-pathogenic *Burkholderia* isolates. Genome Biol. 2010;11(8):R89 doi: 10.1186/gb-2010-11-8-r89 20799932 10.1186/gb-2010-11-8-r89 PMC2945791

[18] Vuddhakul V, Tharavichitkul P, Na-Ngam N, Jitsurong S, Kunthawa B, Noimay P, et al Epidemiology of *Burkholderia pseudomallei* in Thailand. Am J Trop Med Hyg. 1999;60(3):458–61. 10466977 10.4269/ajtmh.1999.60.458

[19] Trakulsomboon S, Vuddhakul V, Tharavichitkul P, Na-Gnam N, Suputtamongkol Y, Thamlikitkul V. Epidemiology of arabinose assimilation in *Burkholderia pseudomallei* isolated from patients and soil in Thailand. Southeast Asian J Trop Med Public Health. 1999;30(4):756–9. 10928371

[20] Ngamdee W, Tandhavanant S, Wikraiphat C, Reamtong O, Wuthiekanun V, Salje J, et al Competition between *Burkholderia pseudomallei* and *Burkholderia thailandensis*. BMC Microbiol. 2015;15(1):56 doi: 10.1186/s12866-015-0395-7 25879538 10.1186/s12866-015-0395-7 PMC4365494

[21] Ngugi SA, Ventura VV, Qazi O, Harding SV, Kitto GB, Estes DM, et al Lipopolysaccharide from *Burkholderia thailandensis* E264 provides protection in a murine model of melioidosis. Vaccine. 2010;28(47):7551–5. doi: 10.1016/j.vaccine.2010.08.058 . 20837078 10.1016/j.vaccine.2010.08.058

[22] Scott AE, Laws TR, D'Elia RV, Stokes MG, Nandi T, Williamson ED, et al Protection against Experimental Melioidosis following Immunization with Live *Burkholderia thailandensis* Expressing a manno-Heptose Capsule. Clin Vaccine Immunol. 2013;20(7):1041–7. Epub 2013/05/17. doi: 10.1128/CVI.00113-13 23677322 10.1128/CVI.00113-13 PMC3697456

[23] Bayliss M, Donaldson MI, Nepogodiev SA, Pergolizzi G, Scott AE, Harmer NJ, et al Structural characterisation of the capsular polysaccharide expressed by *Burkholderia thailandensis* strain E555:: wbiI (pKnock-KmR) and assessment of the significance of the 2-O-acetyl group in immune protection. Carbohydr Res. 2017;452:17–24. doi: 10.1016/j.carres.2017.09.011 29024844 10.1016/j.carres.2017.09.011 PMC5697523

[24] Glass MB, Gee JE, Steigerwalt AG, Cavuoti D, Barton T, Hardy RD, et al Pneumonia and septicemia caused by *Burkholderia thailandensis* in the United States. J Clin Microbiol. 2006;44(12):4601–4. doi: 10.1128/JCM.01585-06 17050819 10.1128/JCM.01585-06 PMC1698378

[25] Wiersinga WJ, Birnie E, Weehuizen TA, Alabi AS, Huson MA, Huis in 't Veld RA, et al Clinical, environmental, and serologic surveillance studies of melioidosis in Gabon, 2012–2013. Emerg Infect Dis. 2015;21(1):40–7. doi: 10.3201/eid2101.140762 25530077 10.3201/eid2101.140762 PMC4285261

[26] Knappik M, Dance DA, Rattanavong S, Pierret A, Ribolzi O, Davong V, et al Evaluation of Molecular Methods To Improve the Detection of *Burkholderia pseudomallei* in Soil and Water Samples from Laos. Appl Environ Microbiol. 2015;81(11):3722–7. doi: 10.1128/AEM.04204-14 25819969 10.1128/AEM.04204-14 PMC4421066

[27] Hantrakun V, Rongkard P, Oyuchua M, Amornchai P, Lim C, Wuthiekanun V, et al Soil Nutrient Depletion Is Associated with the Presence of *Burkholderia pseudomallei*. Appl Environ Microbiol. 2016;82(24):7086–92. doi: 10.1128/AEM.02538-16 27694236 10.1128/AEM.02538-16 PMC5118919

[28] Thailand Demographic [Internet]. www.bora.dopa.go.th. 2013 [cited 23 October 2015]. Available from: http://stat.bora.dopa.go.th/stat/y_stat56.html.

[29] Lands used for agriculture cencus 2013 [Internet]. 2013 [cited 17 July 2015]. Available from: http://www.oae.go.th/download/use_soilNew/soiNew/landused2556.html.

[30] Limmathurotsakul D, Dance DA, Wuthiekanun V, Kaestli M, Mayo M, Warner J, et al Systematic review and consensus guidelines for environmental sampling of *Burkholderia pseudomallei*. PLoS Negl Trop Dis. 2013;7(3):e2105 doi: 10.1371/journal.pntd.0002105 23556010 10.1371/journal.pntd.0002105 PMC3605150

[31] Limmathurotsakul D, Wuthiekanun V, Amornchai P, Wongsuwan G, Day NP, Peacock SJ. Effectiveness of a simplified method for isolation of *Burkholderia pseudomallei* from soil. Appl Environ Microbiol. 2012;78(3):876–7. doi: 10.1128/AEM.07039-11 22101048 10.1128/AEM.07039-11 PMC3264119

[32] Chantratita N, Wuthiekanun V, Boonbumrung K, Tiyawisutsri R, Vesaratchavest M, Limmathurotsakul D, et al Biological relevance of colony morphology and phenotypic switching by *Burkholderia pseudomallei*. J Bacteriol. 2007;189(3):807–17. doi: 10.1128/JB.01258-06 17114252 10.1128/JB.01258-06 PMC1797308

[33] Anuntagool N, Panichakul T, Aramsri P, Sirisinha S. Shedding of lipopolysaccharide and 200-kDa surface antigen during the in vitro growth of virulent Ara- and avirulent Ara+ *Burkholderia pseudomallei*. Acta Trop. 2000;74(2–3):221–8. . 10674653 10.1016/s0001-706x(99)00074-1

[34] Anuntagool N, Naigowit P, Petkanchanapong V, Aramsri P, Panichakul T, Sirisinha S. Monoclonal antibody-based rapid identification of *Burkholderia pseudomallei* in blood culture fluid from patients with community-acquired septicaemia. J Med Microbiol. 2000;49(12):1075–8. doi: 10.1099/0022-1317-49-12-1075 11129718 10.1099/0022-1317-49-12-1075

[35] Duval BD, Elrod MG, Gee JE, Chantratita N, Tandhavanant S, Limmathurotsakul D, et al Evaluation of a latex agglutination assay for the identification of *Burkholderia pseudomallei* and *Burkholderia mallei*. Am J Trop Med Hyg. 2014;90(6):1043–6. doi: 10.4269/ajtmh.14-0025 24710616 10.4269/ajtmh.14-0025 PMC4047727

[36] Nualnoi T, Norris MH, Tuanyok A, Brett PJ, Burtnick MN, Keim PS, et al Development of Immunoassays for *Burkholderia pseudomallei* Typical and Atypical Lipopolysaccharide Strain Typing. Am J Trop Med Hyg. 2017;96(2):358–67. doi: 10.4269/ajtmh.16-0308 27994103 10.4269/ajtmh.16-0308 PMC5303037

[37] Godoy D, Randle G, Simpson AJ, Aanensen DM, Pitt TL, Kinoshita R, et al Multilocus sequence typing and evolutionary relationships among the causative agents of melioidosis and glanders, *Burkholderia pseudomallei* and *Burkholderia mallei*. J Clin Microbiol. 2003;41(5):2068–79. doi: 10.1128/JCM.41.5.2068-2079.2003 12734250 10.1128/JCM.41.5.2068-2079.2003 PMC154742

[38] Ileri SZ. The Indirect Haemagglutination Test in the Diagnosis of Melioidosis in Goats. Br Vet J. 1965;121:164–70. 14306014 10.1016/s0007-1935(17)41254-1

[39] Alexander AD, Huxsoll DL, Warner AR Jr., Shepler V, Dorsey A. Serological diagnosis of human melioidosis with indirect hemagglutination and complement fixation tests. Appl Microbiol. 1970;20(5):825–33. 5530276 10.1128/am.20.5.825-833.1970 PMC377056

[40] Suttisunhakul V, Chantratita N, Wikraiphat C, Wuthiekanun V, Douglas Z, Day NP, et al Evaluation of Polysaccharide-Based Latex Agglutination Assays for the Rapid Detection of Antibodies to *Burkholderia pseudomallei*. Am J Trop Med Hyg. 2015;93(3):542–6. doi: 10.4269/ajtmh.15-0114 26123956 10.4269/ajtmh.15-0114 PMC4559694

[41] Viera AJ, Garrett JM. Understanding interobserver agreement: the kappa statistic. Family medicine. 2005;37(5):360–3. . 15883903

[42] Mukaka MM. Statistics corner: A guide to appropriate use of correlation coefficient in medical research. Malawi Med J. 2012;24(3):69–71. 23638278 PMC3576830

[43] Bursac Z, Gauss CH, Williams DK, Hosmer DW. Purposeful selection of variables in logistic regression. Source Code Biol Med. 2008;3:17 doi: 10.1186/1751-0473-3-17 19087314 10.1186/1751-0473-3-17 PMC2633005

[44] Limmathurotsakul D, Wongsuvan G, Aanensen D, Ngamwilai S, Saiprom N, Rongkard P, et al Melioidosis caused by *Burkholderia pseudomallei* in drinking water, Thailand, 2012. Emerg Infect Dis. 2014;20(2):265–8. doi: 10.3201/eid2002.121891 24447771 10.3201/eid2002.121891 PMC3901481

[45] Levy A, Merritt AJ, Aravena-Roman M, Hodge MM, Inglis TJ. Expanded range of *Burkholderia species* in Australia. Am J Trop Med Hyg. 2008;78(4):599–604. 18385355

[46] Warner JM, Pelowa DB, Gal D, Rai G, Mayo M, Currie BJ, et al The epidemiology of melioidosis in the Balimo region of Papua New Guinea. Epidemiol Infect. 2008;136(7):965–71. doi: 10.1017/S0950268807009429 17714600 10.1017/S0950268807009429 PMC2870883

