# Supplementary material for: Presence of B. thailandensis and B. thailandensis expressing B. pseudomallei-like capsular polysaccharide in Thailand, and their associations with serological response to B. pseudomallei
Source: PLoS Negl Trop Dis. 2018 Jan 24;12(1):e0006193. doi: 10.1371/journal.pntd.0006193 (PMC5809093; doi:10.1371/journal.pntd.0006193)
Supplement: S1 Table — (PDF) [file pntd.0006193.s001.pdf]

Table S1 Number of culture-positive sampling points for *B. pseudomallei* (*B. ps*), *B. thailandensis* (*B. th*) and *B. thailandensis* expressing *B. pseudomallei*-like capsular polysaccharide variant (BTCV) in 61 rice fields in the Northeast (n=21), East (n=19) and Central (n=21) Thailand

**Regions**	**Provinces**	**Field***	**Number of sampling points culture positive for**		
			B. ps	***B. th***	**BTCV**
**Northeast**	Buri Rum	NE1-1	87	0	0
		NE1-2	0	0	0
		NE1-3	97	1	0
	Chaiyaphum	NE2-1	0	0	0
		NE2-2	98	0	0
		NE2-3	0	0	0
	Khonkaen	NE3-1	72	0	0
		NE3-2	94	0	0
		NE3-3	35	0	0
	Udonthani	NE4-1	40	20	0
		NE4-2	0	27	0
		NE4-3	28	0	0
	Nong Bua Lamphu	NE5-1	58	0	0
		NE5-2	0	6	0
		NE5-3	48	7	0
	Loei	NE6-1	4	6	0
		NE6-2	0	0	0
		NE6-3	0	0	0
	Nakhon Ratchasima	NE7-1	2	0	0
		NE7-2	0	0	0
		NE7-3	0	0	0
**East**	Chachoengsao	E1-1	39	29	0
		E1-2	8	70	1
		E1-3	43	33	16
		E1-4	0	24	3
	Prachin Buri	E2-1	10	2	0
		E2-2	6	0	0
		E2-3	0	16	10
	Sa Kaeo	E3-1	17	0	0
		E3-2	0	0	0
		E3-3	81	1	0
	Chanthaburi	E4-1	16	0	0

Regions	Provinces	Field*	Number of sampling points culture positive for		
			B. ps	***B. th***	**BTCV**
		E4-2	1	16	0
		E4-3	0	0	0
	Chon Buri	E5-1	3	0	0
		E5-2	32	0	0
		E5-3	0	44	3
	Rayong	E6-1	1	11	0
		E6-2	0	100	24
		E6-3	57	54	2
**Central**	Phetchabun	C1-1	0	0	0
		C1-2	0	0	0
		C1-3	3	7	0
	Phitsanulok	C2-1	1	74	2
		C2-2	1	70	8
		C2-3	0	0	0
	Pathum Thani	C3-1	0	0	0
		C3-2	0	2	0
		C3-3	0	2	0
	Saraburi	C4-1	0	85	6
		C4-2	0	1	0
		C4-3	0	26	1
	Lop Buri	C5-1	0	0	0
		C5-2	0	0	0
		C5-3	0	0	0
	Nakhon Nayok	C6-1	63	0	0
		C6-2	1	65	0
		C6-3	0	0	0
	Bangkok	C7-1	0	6	0
		C7-2	0	13	0
		C7-3	0	8	0

* Each rice field was divided into a grid system, in which 100 sampling points (10 by 10) were plotted 2.5 m apart. At each sampling point, 10 g of soil at 30 cm depth was collected and cultured for *B. pseudomallei*, *B. thailandensis* and BTCV.
