# Supplementary material for: Presence of B. thailandensis and B. thailandensis expressing B. pseudomallei-like capsular polysaccharide in Thailand, and their associations with serological response to B. pseudomallei
Source: PLoS Negl Trop Dis. 2018 Jan 24;12(1):e0006193. doi: 10.1371/journal.pntd.0006193 (PMC5809093; doi:10.1371/journal.pntd.0006193)
Supplement: S2 Table — (PDF) [file pntd.0006193.s002.pdf]

Table S2 Soil physicochemical properties associated with the presence of *B. thailandensis* in univariable logistic regression models

Soil physicochemical characteristics	Rice fields positive for *B. thailandensis*[1] (n=30)	Rice fields negative for *B. thailandensis*[1] (n=31)	Crude odds ratio (95% confidence interval)	p value
**Physical factors**				
• Sand (%)	13.2 (4.3-78.8)	18.2 (7.8-81.0)	0.98 (0.96-1.01)	0.19
• Silt (%)	34.8 (9.7-56.3)	33.1 (11.1-57.2)	1.03 (0.99-1.07)	0.16
• Clay (%)	46.7 (11.5-58.6)	45.2 (7.3-57.6)	1.01 (0.97-1.05)	0.54
• Moisture (%w/w)	14.6 (2.5-28.3)	12.8 (4.0-0.0)	1.05 (0.96-1.15)	0.32
**Acidity and salinity factors**				
• pH	6.6 (4.9-7.8)	6.8 (5.0-8.1)	0.54 (0.27-1.09)	0.09
• Electrical conductivity (dS/m)	0.3 (0.0-1.5)	0.4 (0.0-1.2)	1.14 (0.28-4.59)	0.85
• Lime requirement (kg/100sqm)	7.5 (0.0-30.0)	5.4 (0.0-23.0)	1.01 (0.95-1.08)	0.73
**Chemical factors**				
• Total nitrogen (mg/kg)	682 (330-2442)	643 (175-1,380)	1.01 (0.99-1.03)[2]	0.10
• Available phosphorous (mg/kg)	8.1 (0.6-28)	5.6 (0.2-38)	1.11 (0.60-2.04)[2]	0.75
• Exchangeable potassium (mg/kg)	57.5 (13.5-184)	58.2 (10.5-252)	0.98 (0.87-1.09)[2]	0.65
• Exchangeable calcium (mg/kg)	808 (494-2,182)	945 (272-4,326)	0.99 (0.99-1.00)[2]	0.11
• Available magnesium (mg/kg)	212 (44-616)	169 (47-823)	1.00 (0.97-1.03)[2]	0.85
• Extractable sulphur (mg/kg)	19 (2.2-114)	12 (0-74)	1.19 (0.96-1.48)[2]	0.12
• Exchangeable sodium (mg/kg)	144 (94-241)	161 (88.5-241)	0.91 (0.79-1.05)[2]	0.21
• Total iron (Fe; g/kg)	24 (1.9-96)	33 (0.6-79.6)	0.98 (0.77-1.24)[2]	0.87
• Total cadmium				
o not detected	19 fields (63%)	17 fields (55%)	1.0	
o detected	11 fields (37%)	14 fields (45%)	0.70 (0.27-1.84)	0.50
• Cation exchange capacity (cmol/mg)	7.6 (0.6-44)	12 (2-48)	0.99 (0.95-1.04)	0.75
**Biological related factors**				
• Organic matter (%w/w)	0.8 (0.2-2.4)	0.9 (0.2-2.8)	0.78 (0.35-1.72)	0.53
• Carbon to nitrogen ratio	6.3 (2.0-22.6)	9.5 (2.3-42.6)	0.92 (0.83-1.02)	0.10

[1] Median (range) unless otherwise specified.

[2] Odds ratio for an increase of 100 mg/kg in nutrient.
