# Supplementary material for: Presence of B. thailandensis and B. thailandensis expressing B. pseudomallei-like capsular polysaccharide in Thailand, and their associations with serological response to B. pseudomallei
Source: PLoS Negl Trop Dis. 2018 Jan 24;12(1):e0006193. doi: 10.1371/journal.pntd.0006193 (PMC5809093; doi:10.1371/journal.pntd.0006193)
Supplement: S3 Table — (PDF) [file pntd.0006193.s003.pdf]

**Table S3 Soil physicochemical properties associated with the presence of *B. thailandensis* expressing *B. pseudomallei*-like capsular polysaccharide (BTCV) in univariable logistic regression models**

| Soil physicochemical characteristics | Rice fields positive for BTCV[1] (n=11) | Rice fields negative for BTCV[1] (n=50) | Crude odds ratio (95% confidence interval) | p value |
|---|---|---|---|---|
| **Physical factors** | | | | |
| • Sand (%) | 14.7 (9.8-42.1) | 15.1 (4.3-81.0) | 0.97 (0.93-1.02) | 0.22 |
| • Silt (%) | 32.3 (29.3-56.3) | 34.6 (9.7-57.2) | 1.03 (0.98-1.09) | 0.27 |
| • Clay (%) | 45.5 (27.3-58.6) | 45.6 (7.3-57.6) | 1.02 (0.97-1.08) | 0.41 |
| • Moisture (%w/w) | 15.4 (10.4-19.5) | 12.9 (2.5-0.0) | 1.05 (0.94-1.18) | 0.35 |
| **Acidity and salinity factors** | | | | |
| • pH | 6.6 (5.1-7.8) | 6.7 (4.9-8.1) | 0.67 (0.29-1.57) | 0.35 |
| • Electrical conductivity (dS/m) | 0.2 (0.1-1.5) | 0.4 (0.0-1.4) | 0.71 (0.11-4.76) | 0.72 |
| • Lime requirement (kg/100sqm) | 7.6 (5.8-22.5) | 6.0 (0.0-30.0) | 1.05 (0.97-1.14) | 0.22 |
| **Chemical factors** | | | | |
| • Total nitrogen (mg/kg) | 792.0 (330-2,442) | 599.9 (175-1,601) | 1.02 (1.00-1.03)[2] | 0.04 |
| • Available phosphorous (mg/kg) | 12.2 (4-28) | 6.1 (0.2-38) | 1.95 (0.95-4.00)[2] | 0.07 |
| • Exchangeable potassium (mg/kg) | 57 (31.7-114.8) | 58 (10.5-252) | 0.93 (0.79-1.10)[2] | 0.41 |
| • Exchangeable calcium (mg/kg) | 841 (696-1,680) | 822 (272-4,326) | 0.99 (0.98-1.00)[2] | 0.28 |
| • Available magnesium (mg/kg) | 193 (123-292) | 174 (44-823) | 0.98 (0.94-1.03)[2] | 0.42 |
| • Extractable sulphur (mg/kg) | 18 (4.3-75) | 18 (0-114) | 1.01 (0.78-1.31)[2] | 0.95 |
| • Exchangeable sodium (mg/kg) | 144 (108-241) | 151 (88.5-241) | 1.02 (0.85-1.22)[2] | 0.83 |
| • Total iron (Fe; g/kg) | 3 (0.4-5.9) | 2.8 (0.1-9.6) | 0.97 (0.71-1.33) | 0.85 |
| • Total cadmium | | | | |
| o not detected | 6 fields (55%) | 30 fields (60%) | 1.0 | |
| o detected | 5 fields (45%) | 20 fields (40%) | 1.25 (0.34-4.65) | 0.74 |
| • Cation exchange capacity (cmol/mg) | 4.7 (0.6-21.9) | 13 (2-48) | 0.92 (0.85-1.00) | 0.05 |
| **Biological related factors** | | | | |
| • Organic matter (%w/w) | 0.8 (0.3-1.5) | 0.9 (0.2-2.8) | 0.47 (0.13-1.61) | 0.23 |
| • Carbon to nitrogen ratio | 5.6 (2.0-11.7) | 9.1 (2.3-42.6) | 0.81 (0.65-1.00) | 0.05 |

[1] Median (range) unless otherwise specified.

[2] Odds ratio for an increase of 100 mg/kg in nutrient.
