# Supplementary material for: Presence of B. thailandensis and B. thailandensis expressing B. pseudomallei-like capsular polysaccharide in Thailand, and their associations with serological response to B. pseudomallei
Source: PLoS Negl Trop Dis. 2018 Jan 24;12(1):e0006193. doi: 10.1371/journal.pntd.0006193 (PMC5809093; doi:10.1371/journal.pntd.0006193)
Supplement: S4 Table — (PDF) [file pntd.0006193.s004.pdf]

Table S4 Soil physicochemical properties associated with the presence of *B. thailandensis* expressing *B. pseudomallei*-like capsular polysaccharide in a multivariable logistic regression model

Soil physicochemical characteristics	Adjusted odds ratio (95% confidence interval)	p value
Total nitrogen (mg/kg)	1.04 (1.01 - 1.07) [1]	0.01
Cation exchange capacity (cmol/mg)	0.86 (0.76 - 0.97)	0.01

[1] Odds ratio for an increase of 100 mg/kg in nutrien
