# Supplementary material for: Presence of B. thailandensis and B. thailandensis expressing B. pseudomallei-like capsular polysaccharide in Thailand, and their associations with serological response to B. pseudomallei
Source: PLoS Negl Trop Dis. 2018 Jan 24;12(1):e0006193. doi: 10.1371/journal.pntd.0006193 (PMC5809093; doi:10.1371/journal.pntd.0006193)
Supplement: S5 Table — (PDF) [file pntd.0006193.s005.pdf]

Table S5. Reports of *B. thailandensis* expressing *B. pseudomallei*-like capsular polysaccharide worldwide from 1921 to 2016.

Year	Strains	Sequence type (allele profile)*	Sources	References
2003	CDC3015869	101 (6-5-9-11-14-20-14)	Blood of a 2-year-old male presenting with drowning, post-cardiac arrest, pneumonia and septicemia in Texas, USA	[38]
2008	E555	696 (6-5-9-11-7-20-14)	Soil in Cambodia	[19]
2012	A-330-05-1-04	696	Water (tap water) in Ubon Ratchathani, northeast Thailand **	[34]
2012-2013	D50	1126 (6-5-9-5-7-7-5)	Soil in Gabon	[37]
2013	ST_10	696	Water in Laos	[39] and personal communication: DABD and Dr Sabine Dittrich
2013-2014	SBXCB001a, SBXCB002a, SBXCB003a, SBXCC001a, SBXCC002a, SBXCC005a, SBXCC008a, SBXCC014b, SBXCC019a, SBXCC020a, SBXPL001a, SBXPL002a, SBXPL005a, SBXPL007a, SBXPL010a, SBXPR001a, SBXPR002a, SBXPR005a, SBXRY001a, SBXRY017a, SBXRY019a, SBXRY030a, SBXRY031a, SBXSR003a, SBXSR004a, SBXSR005a, SBXSR007a	696	Soil in East and Central Thailand	This study

***** The *B. pseudomallei* MLST allele profile corresponds to the gene order *ace-gltB-gmhD-lepA-lipA-narK-ndh* [27].

****** Previously reported as *B. pseudomallei*
